# Validation of a German-language modified Rankin Scale structured telephone interview at 3 months in a real-life stroke cohort

**DOI:** 10.1186/s42466-023-00289-x

**Published:** 2023-11-30

**Authors:** Lennart Steffen Milles, Doreen Pommeranz, Woon Hyung Chae, Jordi Kühne Escolà, Christoph Kleinschnitz, Martin Köhrmann, Benedikt Frank

**Affiliations:** Department of Neurology and Center for Translational Neuro- and Behavioral Sciences (C-TNBS), University Hospital Essen, Hufelandstr. 55, 45147 Essen, Germany

**Keywords:** Modified Rankin Scale, Telephone interview, Ischemic stroke, Outcome assessment, Kappa statistics

## Abstract

**Background:**

The modified Rankin scale (mRS) at 3 months is established as the primary outcome measure in clinical stroke trials. Traditionally, the mRS is assessed through an unstructured face-to-face interview. This approach can be labor-intensive and lead to suboptimal inter-rater reliability. Recently, the Covid-19 pandemic made face-to-face contact even more challenging. To address these issues, we developed and validated a structured German-language questionnaire for mRS testing by telephone.

**Methods:**

In this prospective cohort study, we compared the mRS testing results of the standard face-to-face interview with those obtained in a structured interview by telephone using Cohen’s Kappa.

**Results:**

At our tertiary care stroke center, we included 108 patients who underwent both assessments. In 80.6% of cases (87/108) face-to-face and telephone interview reached identical scores. Linear weighted Kappa was 0.82 (*p* < 0.001). Unweighted Kappa for dichotomized mRS between fair (0–2) and poor (3–6) functional outcome was κ = 0.97 (*p* < 0.001).

**Conclusions:**

Our study validates the use of the German-language structured telephone interview as a reliable instrument for the use in clinical trials. We encourage others to utilize the questionnaire. It is available as an Appendix (Additional file 1) to this publication.

**Supplementary Information:**

The online version contains supplementary material available at 10.1186/s42466-023-00289-x.

## Background

The modified Rankin scale (mRS) is widely used as a measure of functional outcome in clinical stroke trials. Typically, it is assessed through an unstructured interview by a medical professional. [1–3] There is no standardized structure for the interview, even though specific questions are suggested to aid categorization. While the mRS is considered reliable and valid, inter-rater reliability remains a concern [4]. To address this issue, different forms of structured questionnaires have been developed and validated. The results demonstrate improved inter-rater reliability and reduced assessment time. [5–8]

Face-to-face-interviews may be difficult to perform in stroke patients who often suffer from residual disabilities that severely affect their ability to travel [9]. These patients are potentially lost to follow up. In addition, the Covid-19 pandemic recently caused an even larger challenge for face-to-face interviews. In response to these logistical challenges, stroke researchers have turned to telephone assessments, usually with a structured questionnaire. Reliability in comparison with face-to-face interviews was good. [7, 10–12]

One of the most commonly used structured questionnaires was presented by Bruno et al. in 2010. It is easy to use and has been validated for the use by telephone. [6, 13] However, the questionnaire is only available in English. Validated mRS questionnaires are also available in Portuguese, Spanish and Chinese. [8, 11–15]. There are standards available for scientific translation and validation of established questionnaires and testing systems, e.g. PROMIS **(**Patient-Reported Outcomes Measurement Information System standards). [16, 17]. However, there is no universally accepted questionnaire for the mRS. The previously published questionnaires do not provide synonymous or additional questions when patients or caregivers have difficulties understanding. Also, we expected that replicating the results of a traditional unstructured German-language mRS interview would require taking social norms of communication and common misunderstandings into account.

To the best of our knowledge, a validated structured mRS questionnaire in German has not been published. For the reasons mentioned above, instead of testing a translation of another published questionnaire, we developed a short and easy to use structured interview with additional questions and synonyms included. The aim of this study is the validation of our structured mRS telephone questionnaire.

## Methods

In this prospective cohort study, we aimed to validate a German-language structured telephone interview for the mRS by comparing it to the results of the standard face-to-face mRS interview.

**Fig. 1:**
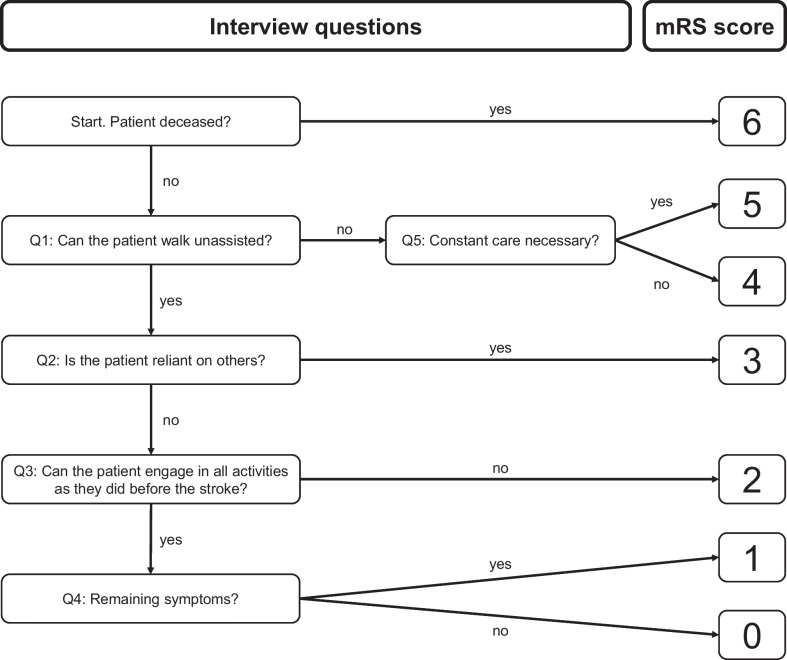
Flowchart describing the course of the structured mRS interview. Q and Numeral (e.g. Q1) = Number of question within the interview. See the questionnaire in the Additional file 1 for a more detailed description

The structured interview was designed in a consensus process involving two stroke experts (BF, MK). It is organized in the form of hierarchical and consecutive questions. With a maximum of 5 questions, the interviewer will reach an mRS score (see interview structure in Fig. 1 and questionnaire in Additional File 1).

For this publication, LSM translated the questionnaire into English. He used a standard online dictionary. [18] It was then reviewed by a native speaker without education in medicine or neurology, because the German version is also written in plain language. The local ethics committee approved the study (Vote 18-840-BO).

Patients were eligible for the study if they (1) had suffered from a stroke 3 months ± 1 month ago, (2) had been treated at our hospital and (3) could be reached by telephone for an interview. Patients with mild cognitive impairments or aphasia were allowed to participate in the study if they were able to return to the hospital for the face-to-face interview and give informed consent.

The standard face-to-face interviews were performed when patients returned for routine follow-up to our clinic. Once the face-to-face interview had been performed, the telephone rater was informed of the patient. She then contacted the patient as soon as possible via telephone to assess with the mRS telephone questionnaire. During the same telephone conversation, routine follow up information was also collected. The telephone and face-to-face interviews were performed by different raters who were blinded for each other’s assessments. The telephone interviews were performed by DP. She was a medical student at the time and received training in stroke patient assessment by telephone mRS by BF and LSM in an one hour session. There was no further communication about scoring patients. DP decided on all mRS scores based on the questionnaire. BF and LSM performed the face-to-face interviews. They are experienced stroke physicians certified to perform the mRS. Patient information and mRS scores were stored in the electronic patient file and later exported for analysis in anonymized form. We performed all statistical tests with SPSS 29 (IBM Corp. Released 2020. IBM SPSS Statistics for Windows, Version 29.0. Armonk, NY: IBM Corp). Scores of the mRS by telephone or face-to-face were compared both as absolute values and for all points of dichotomization within the modified Rankin scale. We performed linear weighted and unweighted Kappa statistics [19]. All statistical tests were two-sided with p = 0.05 as level of significance.

## Results

This study included n = 108 patients with a median age of 69 years (Interquartile range—IQR 59–75 years). Out of the total 108 patients, 49 were female (45.4%). All baseline characteristics are displayed in Table 1. The median mRS at discharge was 1 (IQR 1–2). Median time from hospital admission for acute stroke to telephone interview was 98 days (IQR 92–106 days), median time from admission to face-to-face interview was 97 days (IQR 92–104 days). The median time between face-to-face and telephone interview was 9 days (IQR 3–14 days).

**Table 1:** Baseline characteristics of the n = 108 patients included in this study

Baseline characteristic	
Age (median, IQR)	69 (59–75)
Sex female	49 (45.4%)
Atrial fibrillation	15 (13.9%)
Diabetes	28 (25.9%)
Hypertension	79 (73.1%)
Intracranial bleeding	5 (4.6%)
Ischemic stroke	97 (89.8%)
Transient ischemic attack	6 (5.6%)
NIHSS* on admission (median, IQR)	2 (1–4)
History of stroke	20 (18.5%)
mRS at discharge (median, IQR)	1 (1–2)

^*^NIHSS = National Institute of Health Stroke Scale

Identical scores between telephone and face-to-face-interview were observed in 87 of 108 (80.6%) cases (see Table 2). Unweighted Kappa was 0.73 between telephone interview and face-to-face interview. This value of Kappa is significantly different from zero (κ = 0.73, *p* < 0.001). Weighted Kappa using linear weights was 0.82. This value of Kappa is significantly different from zero (κ = 0.82, *p* < 0.001) (see Table 3). See also Additional file 2: Fig. S1 in the appendix for a visual representation of the mRS distribution.

**Table 2:** Distribution of mRS scores by telephone and face-to-face grouped by score

Modified Rankin Scale face-to-face	Modified Rankin Scale by telephone					Total
	0	1	2	3	4	
0	27	3	3	0	0	33
1	6	30	3	0	0	39
2	0	4	9	1	0	14
3	0	0	0	19	1	20
4	0	0	0	0	2	2
Total	33	37	15	20	3	108

**Table 3:** Analysis of Cohen’s Kappa for the un-dichotomized modified Rankin scale values. Linear weights were used for the weighted Kappa analysis

Kappa method	Kappa value, (95% CI)	*p*-Value	Strength of agreement	Identical scores
Linear weighted	0.82 (0.75–0.90)	< 0,001	very good	87/108 (80.6%)
Unweighted	0.73 (0.63–0.84)	< 0,001	good	87/108 (80.6%)

Regarding the distinction between fair und poor functional outcome, defined as mRS 0–2 vs. mRS 3–6, the telephone interview and face-to-face assessment reached identical scores in 107 of 108 cases (99.1%). Unweighted Kappa was 0.97. This value of Kappa is significantly different from zero (κ = 0.97, *p* < 0.001). Unweighted Kappa was similarly high for all other possible dichotomized analyses of the mRS scale (see Table 4).

**Table 4:** Analysis of Cohen’s Kappa for dichotomized modified Rankin scale values

Dichotomized mRS score	Kappa value, (95% CI)	*p*-Value	Strength of agreement	Identical scores
0 vs. 1–4	0.74 (0.60–0.88)	< 0,001	good	96/108 (88.9%)
0–1 vs. 2–4	0.80 (0.67–0.91)	< 0,001	good	98/108 (90.7%)
0–2 vs. 3–4	0.97 (0.92–1.03)	< 0,001	very good	107/108 (99.1%)
0–3 vs. 4	0.80 (0.41- 1.19)	< 0,001	good	107/108 (99.1%)

The analysis was performed for all possible points of dichotomization within the dataset. As none of the patients was scored as mRS = 5, an analysis of mRS 0–4 vs 5 could not be performed

## Discussion

In this prospective cohort study, we successfully validated the German-language mRS telephone questionnaire in stroke patients at 3 months. We also provide the questionnaire under the creative commons license for public use in the Additional file 1.

The telephone interview and standard face-to-face interview produced similar results. Weighted Kappa using linear weights was 0.82, indicating very good agreement between the two methods. Kappa for the distinction between fair and poor outcome (0–2 vs 3–5) was 0.97 which indicates excellent agreement. To the best of our knowledge, this is the first validated German language mRS telephone questionnaire.

Standardized telephone interviews have been validated in the past for other languages [6, 10, 20]. Previous studies did not assess mRS by telephone at exactly 3 months. In some studies, the index stroke had happened only days before. [7] Assessing the mRS in the hospital setting will likely underestimate the mRS, because patients have not yet experienced their functional deficits in everyday life. Validating the questionnaire at 3 months after the index stroke is a particular strength of our study. It ensures external validity by mimicking the circumstances of mRS assessment in randomized controlled stroke trials.

We hope the results of our study will help to reduce burden for both patients and researchers. We were unable to test whether our telephone mRS assessment was faster than the face-to-face assessment as the latter was part of patients’ routine appointments. Previous studies have shown that a structured questionnaire is more time-efficient. [6] However, the benefit of not having to travel to the hospital for assessment is evident.

We believe our questionnaire is user-friendly because it provides a clear and logical structure aided by additional guidance in the form of alternative questions. In our experience, these alternatives proved valuable when the patient or caregiver had difficulties understanding. In contrast, published questionnaires offer only flow charts or a list of single questions. Notably, the telephone mRS questionnaire was employed by medical student who had only undergone a single training session. This highlights the opportunity for researchers to delegate the mRS assessment to less specialized personnel without compromising accuracy.

The mRS is the standard for clinical stroke trials worldwide. This is not only due to methodical advantages but also the result of its widespread use and acceptance [1].

Our results additionally demonstrate the simplicity of the mRS and highlight the opportunity for valid results without the need for face-to-face contact through assessment by structured interview and thus support the continued use of the modified Rankin Scale in future stroke trials.

The main limitation of our study was the limited number of patients with higher mRS scores, as these patients were unable to return to our outpatient clinic for re-assessment. This is also reflected in the relatively low median NIHSS of 2 in our cohort, which is thus more closely related to that of an ambulatory post-stroke clinic than an acute stroke ward, possibly reducing external validity of our results.

Covid restrictions prevented us from visiting these patients in their homes for study purposes only. Unfortunately, no patient with an mRS of 5 was included.

However, the distinction between scores 4 and 5 is about being fully dependent and bedridden and thus not a challenging one. Additionally, the telephone interviews did identify patients with severe disability. The questionnaire performed best when collapsing the categories of patients with fair vs poor functional outcomes into one category. So, the most important distinction between fair and poor outcome was very reliable in our study.

All patients being interviewed twice might also result in bias in which patients tend to stay with their original answers when being assessed a second time.

## Conclusions

In conclusion, our study validates the German mRS telephone questionnaire for the use in future stroke trials. The structured questionnaire is easy to apply, can be delegated to less specialized personnel and offers comparable performance to the face-to-face assessment. We encourage researchers to utilize this questionnaire for their studies. It is included as Additional file 1 in German and an English translation under the Creative Commons License.

### Supplementary Information

**Additional file 1.** English Translation and Original German-language modified Rankin Scale Telephone Interview.

**Additional file 2. Figure S1.** Comparison of number of patients by mRS score by telephone vs face-to-face.

## Data Availability

The questionnaire is available as Additional file 1 to this publication. The datasets used and analyzed for the current study are available from the corresponding author on reasonable request.

## Abbreviations

mRS: Modified Rankin Scale
IQR: Interquartile range
NIHSS: National Institute of Health Stroke Scale
