# Supplementary material for: Validation of a German-language modified Rankin Scale structured telephone interview at 3 months in a real-life stroke cohort
Source: Neurol Res Pract. 2023 Nov 30;5:59. doi: 10.1186/s42466-023-00289-x (PMC10687899; doi:10.1186/s42466-023-00289-x)
Supplement: Supplementary file 2 — Additional file 2. Figure S1. Comparison of number of patients by mRS score by telephone vs face-to-face. [file 42466_2023_289_MOESM2_ESM.pdf]

Additional File 2: Figure S1

Comparison of number of patients by mRS score by telephone vs face-to-face

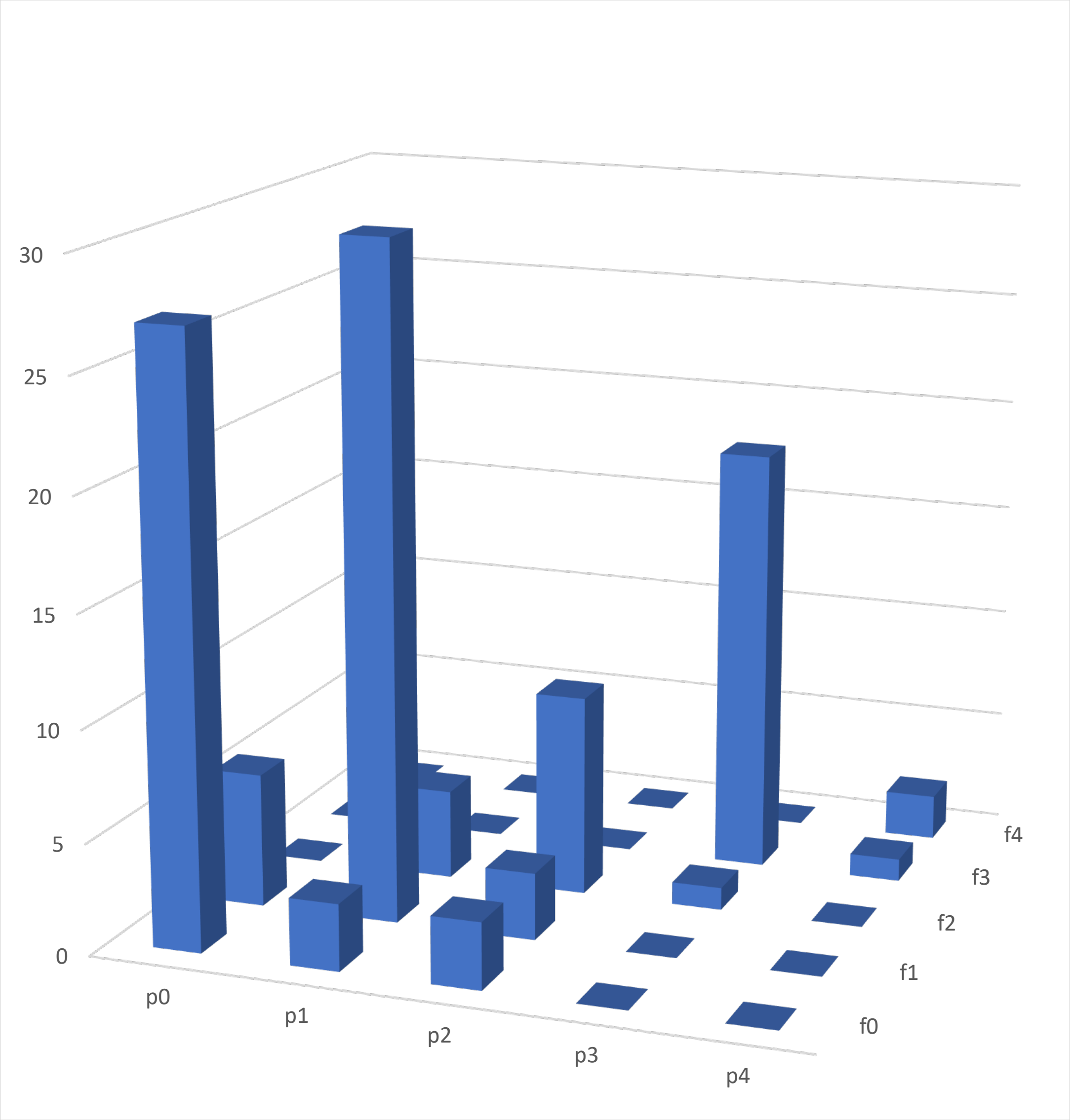

Figure 1: Comparison of the number of patients scored to a specific mRS score by (tele)phone (p0-4) or face-to-face (f0-4) as a 3d bar-chart.
